# A Curriculum Approach to Reduce the Dynamics-Related Reality Gap in Autonomous Driving Decision-Making

**DOI:** 10.3390/s26123734

**Published:** 2026-06-11

**Authors:** Rodrigo Gutiérrez-Moreno, Rafael Barea, Elena López-Guillén, Felipe Arango, Fabio Sánchez-García, Luis M. Bergasa

**Affiliations:** Electronics Department, University of Alcalá (UAH), 28801 Alcalá de Henares, Spain; rafael.barea@uah.es (R.B.); elena.lopezg@uah.es (E.L.-G.); juanfelipe.arango@edu.uah.es (F.A.); fabio.sanchezg@uah.es (F.S.-G.); luism.bergasa@uah.es (L.M.B.)

**Keywords:** autonomous driving, hybrid decision-making, curriculum learning, reality gap, digital twins, parallel execution

## Abstract

Decision-making is a fundamental component of autonomous driving, where complex urban scenarios require safe, robust, and adaptable behaviours. This work presents a curriculum learning approach to reduce the dynamics-related reality gap in autonomous driving decision-making through a hybrid architecture that combines learning-based tactical decisions with classical planning and control methods. The proposed methodology follows a staged sim-to-real process: first, the decision-making policies are trained in a lightweight simulator to learn basic kinematic behaviours; then, they are transferred and refined in CARLA to account for vehicle dynamics; subsequently, a digital twin of the real platform and test environment is used for scenario-specific fine-tuning; finally, the resulting architecture is validated through parallel execution with a real vehicle. The proposed approach focuses on vehicle dynamics, actuation response, and scenario geometry rather than on the complete sim-to-real problem for autonomous driving. The approach is evaluated across multiple urban driving scenarios in simulation, including lane changing, roundabouts, merging, and crossroads, while real-world validation is conducted in a controlled merge scenario. Experimental results show that the proposed curriculum improves training efficiency and final performance across the different stages, achieving success rates above 91% in SMARTS. In CARLA, the proposed architecture completes the evaluated scenarios up to 50% faster than the Autopilot baseline while improving comfort and safety-related metrics in terms of acceleration and jerk. The real-world parallel execution experiment further demonstrates the feasibility of transferring the decision-making architecture to a physical vehicle under controlled conditions. Finally, an ablation study quantifies the contribution of each curriculum stage to the overall system performance.

## 1. Introduction

**Figure 1:**
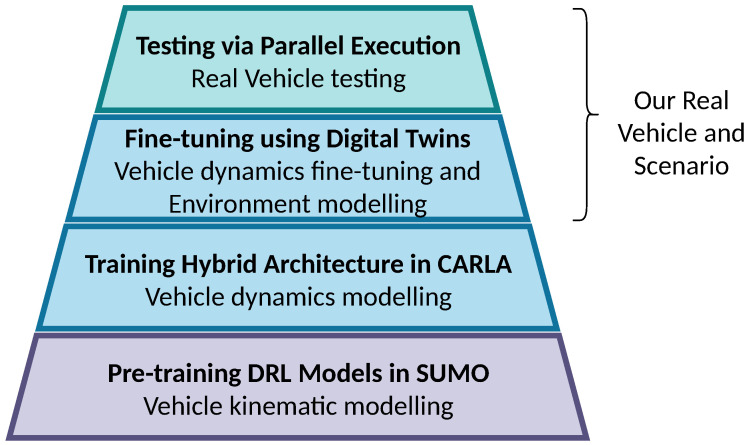
Curriculum methodology and data flow for hybrid decision-making design: (1) tactical policy pre-training in SUMO; (2) policy refinement in CARLA under vehicle dynamics; (3) digital-twin fine-tuning with the real vehicle model and test-scenario geometry; and (4) parallel execution, where the real ego-vehicle state is synchronized with CARLA and simulated adversarial vehicles provide the observations used by the tactical decision-making module.

Integrating Autonomous Driving (AD) systems into urban contexts necessitates a sophisticated Decision Making (DM) framework to analyse complex environmental inputs and perform safe, optimal actions. Recently, Reinforcement Learning (RL) has gained prominence as a state-of-the-art methodology for addressing the intrinsic uncertainties and dynamic complexities characteristic of such environments. However, applying RL training directly to real vehicles can be expensive and unsafe [1]. Hence, safety considerations must not only focus on algorithmic aspects but also consider the costs of sensors and the potential damage to vehicles during the training process. To alleviate these challenges, various strategies have adopted a preliminary experimental stage employing high-fidelity simulations. These simulations replicate critical scenarios, identifying risky behaviours in training the DM system before progressing to real-world tests [2]. Within the field of AD, a notable disparity often arises between simulated environments and real conditions, commonly known as the Reality Gap (RG). Several methodologies have been proposed to overcome this RG, generally falling into three main classifications: (1) Sim2real, which involves transferring knowledge from simulations to the real world, where DM systems are trained in simulation, then refined in real tests; (2) Digital Twins (DT), wherein virtual replicas of the physical world enable vehicles to derive insights regarding their DT through the offline synchronization of data from both real and simulated environments; and (3) Parallel Intelligence (PI) technology, which synthesizes the benefits of Sim2real and DT. Within this paradigm, acquired knowledge is transferred to the physical vehicle via DT, utilizing real-time interaction between physical and virtual domains and online feedback mediated by a Parallel Execution (PE) [2]. In this work, we use the term RG to refer specifically to the dynamics-related discrepancy between simulation and real-world execution, including vehicle motion, actuation response, and scenario geometry. In this work, we present a structured curriculum methodology for the practical implementation and validation of a hybrid DM module in our AD stack, following a Curriculum Learning (CL) strategy and emphasizing four key steps, as depicted in Figure 1.

The first step involves training the tactical learning-based DM policy in a lightweight simulator such as SUMO [3] to develop an initial (kinematic) vehicle behaviour model. In the second step, the trained model is transferred to a hyper-realistic simulation environment (CARLA [4]), performing a second training stage to refine the policy under more realistic vehicle dynamics. The third step involves the building of a DT, which includes the dynamics of our ego vehicle and replicates the road layout of our real testing scenario. This DT serves as a virtual testing setup, allowing our DM approach to be safely evaluated in simulation. Finally, in the fourth step, our DM approach is validated in a real-world setting through a Parallel Execution (PE), where synchronized experiments in both the simulated and physical environments are carried out in real time. Interaction with adversarial vehicles is simulated following an Augmented Reality (AR) strategy, while the framework is evaluated in our real vehicle. This approach narrows the gap between simulated training and real-world application, allowing for greater flexibility in the design of use cases at a low cost and with enhanced safety.

Therefore, this research presents a practical pathway for the development of a hybrid DM architecture that combines learning-based tactical behaviour selection with classical planning and control components for real-world autonomous driving applications. Building upon our prior work [5], we extend our study by introducing four distinct driving scenarios. Additionally, we conduct a deeper analysis of the DRL agents, comparing their performance with that of other State of the Art (SOTA) approaches. The results are also examined more thoroughly, including with respect to comfort metrics. The proposed approach is evaluated across several urban scenarios in simulation, while the PE stage is validated on the real vehicle in a controlled merge scenario. Therefore, the real-world validation should be interpreted as a proof of concept for the proposed transfer pipeline rather than as a complete validation over all urban scenarios. Our key contributions in this work are outlined as follows:

- A structured methodological pipeline for DM design that integrates curriculum learning, DT, and PE into a unified sim-to-real transfer process, with emphasis on the sequencing, integration, and empirical validation of the different stages;
- Empirical validation of the sim-to-real transfer pipeline, demonstrating that policies trained via the proposed curriculum maintain consistent performance when deployed on a real vehicle in a controlled merge scenario, with remaining urban scenarios evaluated in simulation and planned for future real-world validation;
- A comparative evaluation of representative DRL algorithms (DQN, A2C, TRPO, and PPO) within the SMARTS framework, aimed at selecting a suitable tactical policy for the proposed sim-to-real pipeline, providing an engineering benchmark of existing algorithms to identify the most suitable candidates for this specific sim-to-real pipeline;
- Development of a PE system capable of synchronizing real-world vehicle states with a DT in real-time, facilitating the safe testing of AVs against adversarial traffic without physical risk.

This work primarily addresses the dynamics-related component of the RG between simulation and real-world execution. Other relevant aspects of sim-to-real transfer, such as perception uncertainty and complex multi-agent interaction generalization, are outside the scope of the present study and will be considered in future work.

## 2. Related Works

Our survey of DM methodologies for AD revealed a broad spectrum of strategies. Foundational work in this domain relies on classical behavioural models. Prominent examples include the Intelligent Driver Model and leader–follower strategies for longitudinal control, as well as specific lane-change models designed to define safe manoeuvrability trajectories [6]. Furthermore, optimization techniques based on evolutionary algorithms have been explored to enhance manoeuvrability in multi-agent systems [7]. While these classical models remain prevalent and provide essential baselines, this work concentrates on learning-based techniques, an area that has recently garnered substantial interest [8]. Three primary trends are identifiable: (1) Statistical learning frameworks facilitate the acquisition of human-like DM capabilities in ADs via massive datasets [9]. (2) Deep Learning (DL) methods prevail in end-to-end paradigms, employing raw sensor inputs to manage low-level control [10]. (3) RL approaches strive to optimize returns via trial-and-error mechanisms [11]. Of these strategies, RL distinguishes itself as a robust candidate for managing the complexities of DM within the uncertain AD landscape.

Within this domain, DRL has established itself as a premier methodology capable of interpreting environments and deducing optimal choices from input data, thereby exceeding standard RL performance. Implementing DRL necessitates the definition of three core components: the state space, the action space, and the reward mechanism. Concerning state representation, conventional strategies usually prioritize low-dimensional attributes, such as obstacle proximity, lane alignment, or vehicle speed [12]. Such models demonstrate significant robustness and adaptability when facing complex scenarios. Alternative methodologies incorporate high-dimensional inputs, including bird’s-eye-view images [13], image augmentation [14], and occupancy grids [15]. Regarding the action space, certain studies suggest employing high-level directives like “stop”, “drive slow”, or “drive fast” [16], as well as interaction choices like “take way” or “give way” [17]. Others concentrate on lane manoeuvres, utilizing commands such as “change left”, “idle”, and “change right” [18]. With respect to the reward function, positive reinforcement is typically awarded for episode completion, whereas collisions trigger negative penalties. Specifically, ref. [19] correlates positive rewards with vehicle velocity to encourage forward motion. Conversely, ref. [20] imposes a negative penalty proportional to the simulation duration of each episode. While these methods address particular scenarios, they yield only fractional solutions when scaled to broader applications.

Conversely, several studies introduce comprehensive AD implementations grounded in DRL. For instance, one approach utilizes a Scene-Rep Transformer to augment the capabilities of RL DM [21]. This method defines actions as the ego vehicle’s longitudinal velocity and lane-change signals, which are executed via the SUMO simulator. Other research places greater emphasis on realistic integration within an AD architecture. Notably, the authors of [22] proposed an attention-driven driving policy designed to manage unprotected intersections using DRL. Furthermore, a hybrid strategy is presented in [23], describing a DM and control framework that leverages the advantages of both rule-based and learning-based techniques while mitigating their respective drawbacks. While these proposals cover diverse scenarios and approach real-world utility, they face significant obstacles regarding extendibility beyond virtual environments. Consequently, the majority of research involving DRL-based methods remains confined to simulated experiments.

Nevertheless, this proposal targets operations with real vehicles, where safety and cost are critical considerations. Thus, the transition from the simulated domain to the real world is paramount. To bridge this RG, as previously noted, three distinct strategies are examined. Concerning Sim2Real techniques [24], CL functions as a training strategy progressing from simple to complex data, mirroring the learning sequence of human curricula [25]. In [19], an automatic curriculum generation method is proposed, while ref. [26] secures improved overtaking performance via a three-stage CL methodology. Transfer learning provides another avenue, where knowledge learned from one task is repurposed to boost performance on a related task. Ref. [27] validates that transfer learning utilizing simulated accident data improves generalization across diverse scenarios. Similarly, ref. [28] executes transfer learning for semantic segmentation in off-road environments using a pre-trained network. An alternative approach is the use of a DT, a digital replica of a physical entity capable of simulating the system’s entire lifecycle and synchronizing with the physical twin [29]. A strategy to efficiently train a DRL policy in simulation for deployment in a real-time vehicle is shown in [30]. In [31], a DT environment model predicting physical transition dynamics is proposed to enhance RL data efficiency, which often requires extensive agent–environment interactions during training. Recently, researchers have adopted PI approaches to facilitate knowledge transfer from simulation to reality. These methods combine the strengths of Sim2real and DT in modelling complex systems, addressing the challenges individual methods face regarding the RG. Liu et al. [32] improved intelligent vehicle safety by integrating virtual vehicles with diverse roles into complex physical scenarios. Wang et al. [33] introduced the core concept of parallel testing, utilizing a cyclic updating method to resolve the RG problem.

Despite the significant progress made by these studies, a conspicuous gap remains regarding comprehensive real-world implementations that seamlessly integrate RL-based DM systems within a complete vehicle architecture to effectively bridge the RG. Addressing this limitation, our approach presents a concrete pathway for integrating a learning-based tactical decision module within a complete hybrid AD architecture and for progressively transferring it from simulation to real-world execution. Furthermore, we corroborate the practical applicability of this methodology by moving beyond virtual validation and conducting experimental testing on a physical vehicle.

## 3. Background

Our proposal treats the tactical DM module as a learning-based decision process under partial observability, using Partially Observable Markov Decision Process (POMDP) notation to define states, observations, actions, and rewards. This section establishes the theoretical groundwork for our implementation by defining these concepts.

### 3.1. POMDP Formulation

A POMDP extends the classical Markov Decision Process (MDP) framework by considering that the agent does not have full access to the real state of the environment. It can be formally defined by the tuple expressed as (S,A,T,R,Ω,O), where *S* is the state space, *A* is the action space, *T* is the transition function, *R* is the reward function, Ω is the observation space, and *O* is the observation function.

Due to partial observability, the agent cannot directly access the true state of the environment. Classical POMDP solutions usually maintain a belief state, defined as a probability distribution over possible states. However, in this work, no explicit belief distribution, Bayesian filter, recurrent network, or observation-history mechanism is implemented. Instead, the problem is treated as DRL under partial observability, where the policy acts according tothe instantaneous low-dimensional observation provided by the active scenario.

### 3.2. Deep Reinforcement Learning

In RL, the agent learns a policy (π) that selects an action (*a*) from the information available at each time step, with the objective of maximizing the accumulated reward obtained through interaction with the environment. In fully observable MDPs, the transition process satisfies the Markov property, i.e.,

(1) $$P\left(s_{t+1}|s_{t},s_{t-1},\ldots ,s_{0},a_{t}\right)=P\left(s_{t+1}|s_{t},a_{t}\right).$$

In this partially observable setting, the true state (*s*) is not directly available, and the agent receives an observation (*o*) instead. Consequently, the DM process is based on the information contained in the current observation rather than on an explicitly updated belief state. Deep reinforcement learning addresses this problem by using Deep Neural Networks (DNNs) to approximate the policy, the value function, or both. In the proposed architecture, the learned policy is used at the tactical level. During training, the policy or value-function approximator is updated to increase the expected reward. For policy-gradient methods, this update can be expressed through the following general objective:

(2) $$L^{PG}\left(\theta \right)={\hat{E}}_{t}\left[\log\pi_{\theta }\left(a_{t}|s_{t}\right){\hat{A}}_{t}\right],$$

where $\pi_{\theta }$ is the policy and ${\hat{A}}_{t}$ is an estimator of the advantage function.

### 3.3. Deep Reinforcement Learning Algorithms

In this work, we evaluate representative DRL algorithms from both value-based and policy-based families. Specifically, we consider Deep Q-Network (DQN) as a value-based method [34] and Advantage Actor–Critic (A2C), Trust-Region Policy Optimization (TRPO), and Proximal Policy Optimization (PPO) as policy-based actor–critic methods [35,36,37]. These algorithms are selected as established baselines to identify a suitable DM policy for the proposed curriculum-based sim-to-real pipeline.

## 4. Curriculum Methodology

Urban driving environments encompass a variety of scenarios. We formulate these scenarios using POMDPs, treating each of them independently. This method allows for a segmented understanding of the DM process, breaking it down into distinct tasks. The vehicle under control in each scenario is defined as an agent, with one agent for each scenario. Building on the concepts introduced in the previous section, an agent gathers data from the environment in the form of observations and executes actions based on a defined policy. This policy is updated through the training process using the reward function. The development of the training and testing of the agents are done in a curricular way, organized into four key phases: pre-training of kinematic models in SUMO, integration and training of dynamic models in CARLA, DT design with vehicle and environment fine-tuning, and real-vehicle validation through PE. In each curriculum stage, vehicle motion and environmental evolution are provided by the corresponding simulation environment rather than being manually defined by our method. Thus, SUMO provides a simplified traffic-level and kinematic evolution for efficient pre-training, whereas CARLA provides a physics-based dynamic vehicle response for policy refinement. The role of the proposed methodology is to transfer and fine-tune the DM policy across these environments rather than to explicitly model vehicle dynamics. The curriculum is defined by staged transfer across increasingly realistic domains rather than by changes in the neural network architecture. The same policy/value network structure is maintained across the SUMO, CARLA, and DT stages, while the learned weights are progressively reused and fine-tuned. The purpose of increasing simulator fidelity is not to change the policy architecture but to retrain the tactical policy under progressively more realistic closed-loop transition dynamics produced by the simulator and the operative control layer.

### 4.1. Pre-Training in SUMO

**Figure 2:**
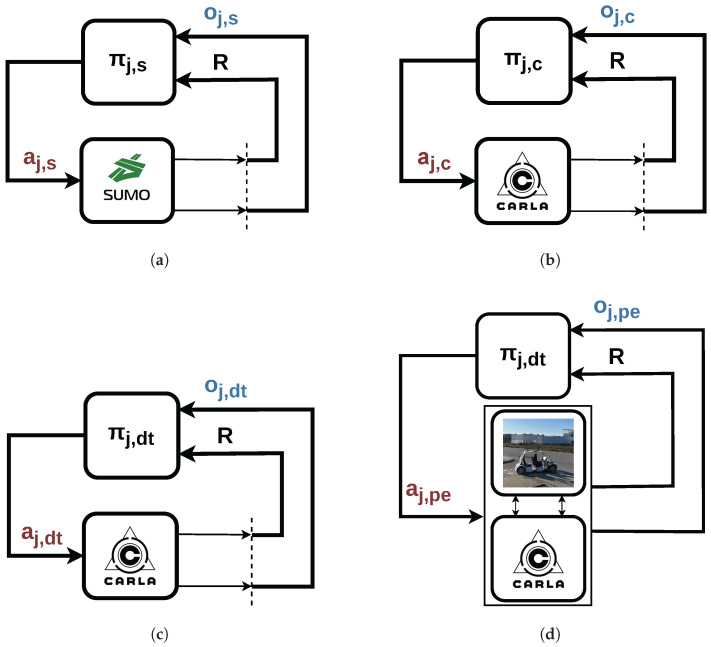
A visual representation of the curriculum methodology: (**a**) Training basic behaviour without vehicle dynamics in SUMO. (**b**) Re-training in CARLA with vehicle dynamics. (**c**) Fine-tuning the DRL models for the real experimental setup using digital twins. (**d**) Validating our approach through a parallel execution.

The initial phase uses SUMO for its fast simulation capabilities, allowing for training at a low computational cost. Although SUMO lacks vehicle dynamics, it provides a sufficient environment for the agent to learn kinematic behaviours and shape an initial policy. Here, the policy weights are randomly initialized, and each agent interacts with its scenario-specific environment to learn an appropriate policy. Therefore, as depicted in Figure 2a, the agent’s action ($a_{j,s}$) is taken based on the policy ($\pi_{j,s}$), which depends on the observation ($o_{j,s}$) and the learned weights ($\theta_{j,s}$):

(3) $$a_{j,s}=\pi_{j,s}\left(o_{j,s}|\theta_{j,s}\right)$$

The observation ($o_{j,s}$) that is provided to the agent is derived from the action that is taken ($a_{j,s}$) and the transition function ($T_{j,s}$):

(4) $$o_{j,s}=f_{j,s}\left(a_{j,s},T_{j,s}\right)$$

Here, *j* represents the driving scenario, while *s* indicates that this simulation phase is conducted in SUMO.

### 4.2. Training in CARLA

Once kinematic behaviours are learned, the agent undergoes a second training phase in CARLA, which provides a realistic simulation of vehicle dynamics by using a generic agent. Training from scratch in this high-fidelity simulator could be time-intensive and has a risk of non-convergence, as discussed in our previous work [38]. In this second phase, transfer learning is implemented by initializing the CARLA policy/value networks with the weights learned in the previous SUMO stage. The network architecture remains unchanged, and the parameters are updated through additional training episodes in CARLA under the new dynamic vehicle model and simulation conditions. Therefore, the CARLA weights ($\theta_{j,c}$) are obtained by fine-tuning the SUMO-trained weights ($\theta_{j,s}$) rather than by reinitializing the model from scratch. As illustrated in Figure 2b, the agent’s action ($a_{j,c}$) is determined based on the policy ($\pi_{j,c}$), which depends on the observation ($o_{j,c}$) and the fine-tuned weights ($\theta_{j,c}$).

(5) $$a_{j,c}=\pi_{j,c}\left(o_{j,c}|\theta_{j,c},\theta_{j,s}\right)$$

Again, the observation ($o_{j,c}$) that is provided to the agent is influenced by the action that is taken ($a_{j,c}$) and the transition function ($T_{j,c}$):

(6) $$o_{j,c}=f_{j,c}\left(a_{j,c},T_{j,c}\right)$$

Here, *c* indicates that the simulation is carried out by CARLA. These equations describe policy evaluation and observation construction during the CARLA training stage. They are not intended to define an additional recursive dynamical model. The temporal evolution of the scenario is handled by the CARLA simulator through its internal dynamics, while the transfer from SUMO to CARLA is represented by initializing and fine-tuning the policy/value network weights from $\theta_{j,s}$ to $\theta_{j,c}$.

### 4.3. Fine-Tuning Using a Digital Twin

As a preliminary step toward real-world implementation, a DT of our vehicle and environment is created. We first obtain our vehicle’s physical parameters and take measurements of our real test scenario to accurately replicate them in CARLA. Once our vehicle dynamics and testing-environment models are established, we start a new training phase to generate the DRL models in simulation for our real experiments. This training process begins with the previously acquired weights from CARLA $\theta_{j,c}$, which serve as prior information. Subsequently, we retrain with the DT to obtain a new policy ($\pi_{j,dt}$). As illustrated in Figure 2c, the agent’s action ($a_{j,dt}$) is determined by the policy ($\pi_{j,dt}$), which depends on the observation ($o_{j,dt}$) and the weights from CARLA ($\theta_{j,c}$) fine-tuned for the DT ($\theta_{j,dt}$).

(7) $$a_{j,dt}=\pi_{j,dt}\left(o_{j,dt}|\theta_{j,dt},\theta_{j,c}\right)$$

Furthermore, the observation ($o_{j,dt}$) provided to the agent is a function of the action that is taken ($a_{j,dt}$) and the transition function ($T_{j,dt}$):

(8) $$o_{j,dt}=f_{j,dt}\left(a_{j,dt},T_{j,dt}\right)$$

In these equations, dt denotes that the agent operates within the DT, mimicking our environment and the dynamics of our real platform.

### 4.4. Parallel Execution

The final phase of our methodology is a PE to validate the applicability of our architecture in a real-world test. Here, the agent executes actions according to the previously learned policy ($\pi_{j,dt}$). The actions during this phase are produced as follows:

(9) $$a_{j,pe}=a_{j,dt}=\pi_{j,dt}\left(o_{j,dt}|\theta_{j,dt},\theta_{j,c}\right)$$

In this phase, the ego vehicle operates in the real world, interacting with its physical environment and responding to real dynamics. However, adversarial vehicles are represented within a simulated environment in CARLA, allowing for controlled testing scenarios without introducing real-world adversarial agents. This approach combines the real-world dynamics ($T_{j,rw}$) of the ego vehicle with the virtual dynamics ($T_{j,dt}$) of simulated adversaries, creating a mixed-reality environment. The observations provided to the agent depend on both the simulation in CARLA and the real world:

(10) $$o_{j,pe}=f_{j,pe}\left(a_{j,pe},T_{j,rw},T_{j,dt}\right)$$

Here, pe denotes Parallel Execution, while rw refers to the real world.

## 5. Our Architecture

**Figure 3:**
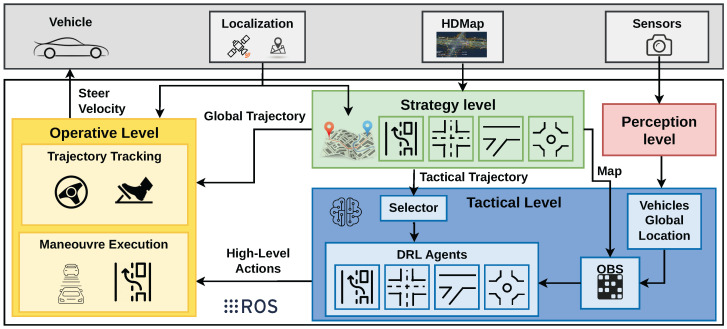
The proposed hybrid DM architecture: strategy, tactical and operative levels.

We carry out the curricular methodology using our AD stack, as previously detailed in [39]. This approach comprises four distinct levels (Figure 3): perception, strategy, tactical, and operative levels.

While the perception level handles the processing of sensor data, it is not the main focus of this work. For this reason, the surrounding vehicles used by the DM module are obtained from the simulator ground truth. The other three levels—namely, the strategy, tactical, and operative levels—constitute the hybrid DM architecture evaluated in this work.

The strategy level [40] consists of a global planner and a scenario planner. The global planner defines the route to be followed by the ego vehicle using the HD map and vehicle localization. The scenario planner identifies the relevant driving situations along this route, such as lane changes, merges, roundabouts, and crossroads. Thus, scenario segmentation and switching are handled by the strategy level rather than by the POMDP formulation or by a single DRL policy. This also means that the road geometry of each use case is implicitly defined by the selected scenario, the HD map, and the reference route.

The tactical level receives the active scenario, HD map information, the ego-vehicle state, and information about surrounding vehicles. Based on these inputs, the corresponding scenario-specific DRL agent is selected. The output of this level is a high-level discrete decision. In this work, these decisions are defined as *drive*, *stop*, *change left*, and *change right*, depending on the active scenario. Therefore, the learning-based component is restricted to tactical behaviour selection, while continuous motion generation remains explicitly handled by the operative layer.

The above decisions are executed by the operative level through a linear quadratic regulator [41] for nominal trajectory tracking and a model predictive control layer [42] for manoeuvre execution.

### 5.1. Operative Execution of Tactical Actions

**Figure 4:**
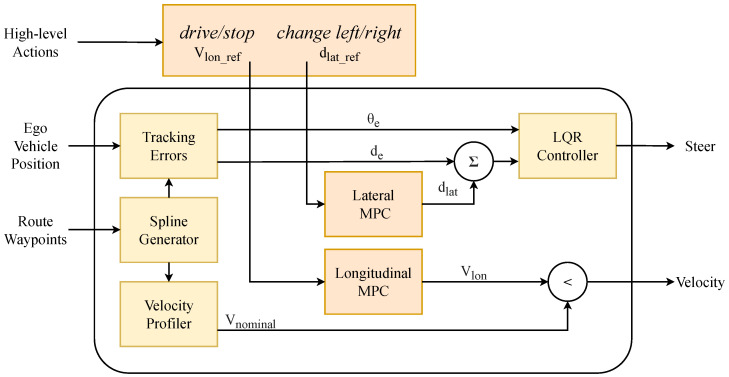
Interface between tactical decisions and operative control.

The operative level follows the hybrid control architecture introduced in [42] and summarized in Figure 4. The tactical module does not produce low-level actuation commands directly. Instead, each discrete action is treated as a manoeuvre request that modifies the longitudinal and/or lateral references of the operative controller.

In nominal driving, the route waypoints are interpolated by the spline generator. The velocity profiler computes the nominal velocity ($v_{\mathrm{nom}}$) from the path curvature, and the LQR controller computes the steering command from the tracking errors ($d_{e}$ and $\theta_{e}$). Tactical actions enter the controller through two MPC references: the longitudinal velocity reference ($v_{\mathrm{lon},\mathrm{ref}}$) and the lateral offset reference ($d_{\mathrm{lat},\mathrm{ref}}$). For the *drive* action, the vehicle follows the nominal velocity profile and keeps $d_{\mathrm{lat},\mathrm{ref}}=0$. For the *stop* action, $v_{\mathrm{lon},\mathrm{ref}}=0$, and the distance to the conflict point is used as the longitudinal bound ($D_{\mathrm{front}}$). For lane-change actions, $d_{\mathrm{lat},\mathrm{ref}}\in \left\{-L_{w},0,L_{w}\right\}$, where $L_{w}$ is the lane width and the sign depends on the target lane.

Let χ=(x,y,θ) be the ego-vehicle pose and $\chi_{d}=\left(x_{d},y_{d},\theta_{d}\right)$ be the closest reference pose on the spline trajectory. The LQR controller tracks the nominal path by minimizing the lateral and heading errors, i.e.,

(11) $$\xi =\left[\begin{array}{c} d_{e}\\ \theta_{e} \end{array}\right]=\left[\begin{array}{c} \left(y-y_{d}\right)\cos\left(\theta_{d}\right)-\left(x-x_{d}\right)\sin\left(\theta_{d}\right)\\ \theta -\theta_{d} \end{array}\right],$$

and by applying the control law, i.e.,

(12) $$\rho_{\mathrm{cmd}}=-K\left[\begin{array}{c} d_{e}+d_{\mathrm{lat}}\\ \theta_{e} \end{array}\right],$$

where *K* is the LQR gain and $d_{\mathrm{lat}}$ is the lateral offset generated by the lateral MPC. Lane-change manoeuvres are therefore introduced as reference offsets.

For longitudinal manoeuvres, the MPC uses a jerk-based triple-integrator model:

(13) $$X_{\mathrm{lon}}\left(k+1\right)=\left[\begin{array}{ccc} 1 & T_{s} & \frac{T_{s}^{2}}{2}\\ 0 & 1 & T_{s}\\ 0 & 0 & 1 \end{array}\right]X_{\mathrm{lon}}\left(k\right)+\left[\begin{array}{c} \frac{T_{s}^{3}}{6}\\ \frac{T_{s}^{2}}{2}\\ T_{s} \end{array}\right]j_{\mathrm{lon}}\left(k\right),\;X_{\mathrm{lon}}={\left[\begin{array}{ccc} d_{\mathrm{lon}} & v_{\mathrm{lon}} & a_{\mathrm{lon}} \end{array}\right]}^{T}.$$

The longitudinal bounds limit the travelled distance, velocity, acceleration, and jerk as

(14) $$\begin{array}{cc} 0 & \leq d_{\mathrm{lon}}\leq D_{\mathrm{front}},\\ 0 & \leq v_{\mathrm{lon}}\leq v_{\mathrm{nom}},\\ a_{\mathrm{lon},\min} & \leq a_{\mathrm{lon}}\leq a_{\mathrm{lon},\max},\\ -|j_{\max}| & \leq j_{\mathrm{lon}}\leq \left|j_{\max}\right|. \end{array}$$

For lateral manoeuvres, the MPC uses a double-integrator model:

(15) $$X_{\mathrm{lat}}\left(k+1\right)=\left[\begin{array}{cc} 1 & T_{s}\\ 0 & 1 \end{array}\right]X_{\mathrm{lat}}\left(k\right)+\left[\begin{array}{c} \frac{T_{s}^{2}}{2}\\ T_{s} \end{array}\right]a_{\mathrm{lat}}\left(k\right),\;X_{\mathrm{lat}}={\left[\begin{array}{cc} d_{\mathrm{lat}} & v_{\mathrm{lat}} \end{array}\right]}^{T}.$$

The lateral bounds constrain the offset around the target lane while limiting lateral velocity and acceleration:

(16) $$\begin{array}{cc} d_{\mathrm{lat},\mathrm{ref}}-\frac{L_{w}-V_{w}}{2} & \leq d_{\mathrm{lat}}\leq d_{\mathrm{lat},\mathrm{ref}}+\frac{L_{w}-V_{w}}{2},\\ -|v_{\mathrm{lat},\max}| & \leq v_{\mathrm{lat}}\leq \left|v_{\mathrm{lat},\max}\right|,\\ -|a_{\mathrm{lat},\max}| & \leq a_{\mathrm{lat}}\leq \left|a_{\mathrm{lat},\max}\right|, \end{array}$$

where $V_{w}$ is the vehicle width. The MPC modules are solved as a constrained quadratic problem:

(17) $$\min_{j_{\mathrm{lon}},a_{\mathrm{lat}}}\Phi \left(X\left(k\right),u\left(k\right)\right)\;s.t.\;h_{\mathrm{lon}}\left(k\right),h_{\mathrm{lat}}\left(k\right),$$

with

(18) $$\Phi \left(X\left(k\right),u\left(k\right)\right)={\left(d_{\mathrm{lat}}\left(k\right)-d_{\mathrm{lat},\mathrm{ref}}\left(k\right)\right)}^{2}+{\left(v_{\mathrm{lon}}\left(k\right)-v_{\mathrm{lon},\mathrm{ref}}\left(k\right)\right)}^{2}.$$

The longitudinal output ($v_{\mathrm{lon}}$) is combined with the nominal velocity by selecting the most restrictive command, as shown by the lower-value block in Figure 4:

(19) $$v_{\mathrm{cmd}}=\min\left(v_{\mathrm{nom}},v_{\mathrm{lon}}\right).$$

The outputs of the operative level are the target linear velocity ($v_{\mathrm{cmd}}$) and the steering angle ($\rho_{\mathrm{cmd}}$), which are sent either to the simulator interface or to the real vehicle drive-by-wire module.

### 5.2. Deep Reinforcement Learning Architecture

**Figure 5:**
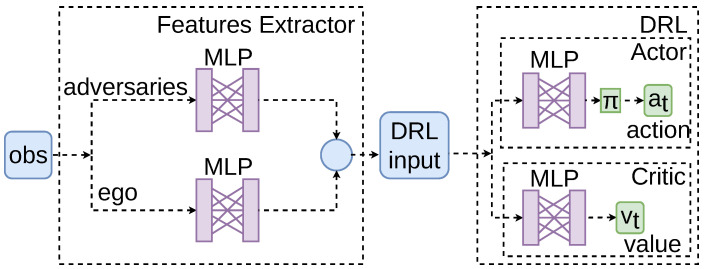
Policy-based algorithm configuration.

Focusing on the tactical level, several DRL agents are implemented, sharing the same architecture as shown in Figure 5, which is divided into two main components:

Feature Extractor Module: In line with insights from our previous research, this work incorporates a feature extraction module, which has proven to enhance the convergence of training [38]. It comprises a dense Multi-Layer Perceptron (MLP) that processes observations from the environment. Information pertaining to both adversarial and ego vehicles is separately processed through the feature extractor, then concatenated into a single vector, serving as the input for the DRL algorithms.

DRL Algorithms: Our study employs two categories of algorithms: value-based (DQN) and policy-based (A2C, TRPO, and PPO) algorithms. The value-based algorithm incorporates a single MLP for its operation, in contrast to the policy-based algorithms, which adopt an actor–critic framework. Within this framework, one MLP functions as the actor, determining the actions to take, while a separate MLP serves as the critic, evaluating the value of the selected action.

All MLPs have two hidden layers, with each layer comprising 128 neurons, and utilize the *tanh* activation function. The input layer’s dimension is based on the number of elements in the observations. The dimension of the output layer corresponds to the number of possible actions. This architecture is kept fixed throughout all curriculum stages. Therefore, differences between stages do not arise from changes in the policy or value networks but from the training environment, the vehicle dynamics model, and the initialization of the network weights.

### 5.3. POMDP Modelling for Urban Scenarios

**Figure 6:**
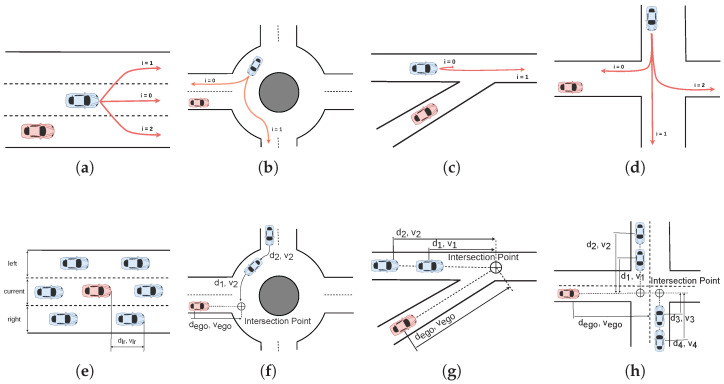
State and observation representations of the scenarios. (**a**) Lane-change state. (**b**) Roundabout state. (**c**) Merge state. (**d**) Crossroad state. (**e**) Lane-change observations. (**f**) Roundabout observations. (**g**) Merge observations. (**h**) Crossroad observations.

We identify and explore four key scenarios that are common in many cities: different types of intersections (crossroads, merges, and roundabouts) and lane change. Each scenario is handled by the strategic scenario planner, then modelled independently at the tactical level using a uniform POMDP formulation. In this context, the POMDP formulation is not used for scenario segmentation but to represent the partial observability of the DM problem within each selected scenario. This formulation is characterized by low-dimensional observation vectors and high-level actions (see Figure 6). Specifically, we define the scenarios as follows:

#### 5.3.1. State Space

The state of a vehicle is defined by its distance to a relevant point ($d_{i}$), its longitudinal velocity ($v_{i}$), and its driving intention ($i_{i}$: $s_{i}=\left[d_{i},v_{i},i_{i}\right]\in R\times R\times \left\{0,1,2\right\}$). For the lane-change scenario, $d_{i}$ is the distance of each adversarial to the ego vehicle, and each vehicle has three possible intentions—change left (i=1), keep driving in its lane (i=0), or change right (i=2)—as illustrated in Figure 6a. In intersection scenarios, $d_{i}$ represents the distance to the intersection point, and driving intentions depend on the specific type of intersection. In a roundabout, the intention is predefined by the route to be followed, where vehicles exit at the first (i=0) or second exit (i=1), as shown in Figure 6b. In the merge scenario, adversarial vehicles are divided according to their level of cooperation: vehicles that consistently yield (i=0) or those that proceed without yielding (i=1). These behaviours are illustrated in Figure 6c. Finally, for crossroads, intentions correspond to the intended route. Vehicles turning right, going straight, and turning left are represented by i=0, i=1, and i=2, respectively. These intentions are illustrated in Figure 6d.

#### 5.3.2. Observation Space

The observation function maps the complete simulator state to the partial input vector used by the policy network. In this work, this mapping mainly consists of selecting the relevant surrounding vehicles and removing non-observable intention variables from the input representation. For instance, for a vehicle state defined as $s_{i}=\left[d_{i},v_{i},i_{i}\right]$, the corresponding observation provided to the policy is $o_{i}=\left[d_{i},v_{i}\right]$. In our approach, vehicles are not able to know the intentions of surrounding vehicles. Therefore, the observation of a vehicle is defined by its distance to a relevant point ($d_{i}$) and its longitudinal velocity ($v_{i}$: $o_{i}=\left[d_{i},v_{i}\right]\in R\times R$). Specifically, in the lane-change scenario, the observation matrix is defined by the nearest vehicles in the current and adjacent lanes relative to the ego vehicle. As shown in Figure 6e, we consider the information of six vehicles: three leading vehicles and three following vehicles. The observation is represented as $\Omega =(d_{ll},v_{ll},d_{lc},v_{lc},d_{lr},v_{lr},d_{fl},v_{fl},d_{fc},v_{fc},d_{fr},v_{fr})$. In the roundabout scenario, the observation vector is defined as $\Omega =(d_{e},v_{e},d_{1},v_{1},d_{2},v_{2})$, where $d_{e}$ and $v_{e}$ refer to the ego vehicle’s position and velocity and $d_{1},v_{1},d_{2},v_{2}$ correspond to the two nearest adversarial vehicles. The vector is structured such that the closest relevant vehicle always occupies the first position, as illustrated in Figure 6f. In the merge scenario, the observation vector is defined in the same way, as shown in Figure 6g. Finally, in the crossroad scenario, where more than two vehicles are considered, the observation vector is defined as $\Omega =(d_{e},v_{e},d_{1},v_{1},\ldots ,d_{4},v_{4})$, encompassing the observations of the ego vehicle and the two closest adversarial vehicles in each lane, as represented in Figure 6h.

#### 5.3.3. Action Space

Our DM system has four possible actions—drive, stop, change left, and change right—so we propose a discrete set of actions for each scenario. In the lane-change scenario, the action space is determined by three high-level actions: ‘change left’, ‘continue straight’, and ‘change right’. These actions can be executed at any time while the agent is driving on a road with more than one lane. The action space in this scenario is defined as follows: a=(changeleft,drive,changeright). Furthermore, the action space for navigating intersections consists of two high-level actions: ‘stop’ and ‘drive’. These actions are strategically executed before entering the intersection and are designed to guide the vehicle as to when to merge into the intersection and when to yield to other vehicles. The action space for this system is thus defined as follows: a=(stop,drive).

#### 5.3.4. Reward Function

The objective of an RL algorithm is to optimize the expected value of the discounted future reward. The purpose of the reward function in these use cases is to perform the safest navigation of the ego vehicle through a scenario, avoiding collisions with adversarial vehicles. Collisions result in a negative reward, while successful navigation results in a positive reward. To further encourage the vehicle’s forward progression, a small cumulative reward based on longitudinal velocity is proposed. Additionally, at the end of each episode, a small negative reward is assigned proportionally to its duration, where *t* represents the episode’s duration and $t_{out}$ represents the timeout time. The function is defined by a reward based on the velocity ($k_{v}\cdot v_{ego}$), a reward for crossing the intersection (+1), a penalty for collisions (−2), and a penalty relative to the episode’s duration ($-0.2t/t_{out}$). Here, $k_{v}=2\times 10^{-3}$. Under this setup, the episode reward falls within the range of [−2,1.1].

### 5.4. Parallel Execution Implementation

**Figure 7:**
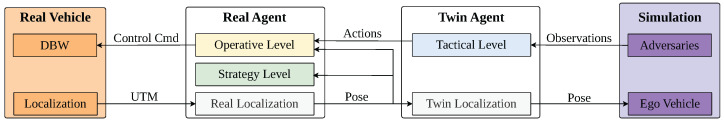
Parallel execution data flow. The Real Agent receives the ego-vehicle localization and sends it to the Twin Agent, which updates the corresponding ego-vehicle pose in CARLA. Simulated adversarial vehicles in CARLA generate the surrounding-traffic observations used by the decision-making module. The selected high-level action is then sent back to the Real Agent and executed by the operative layer through the DBW module.

To bridge the gap between simulation and real-world applications, we develop an agent capable of translating the vehicle’s movements from the real environment into the simulation and actions from the simulator to the real vehicle. This approach enables decisions obtained from the simulated environment to be applied directly to a physical vehicle, thereby facilitating a seamless transition from virtual to real-world testing. The real vehicle is mirrored in the simulator, and the simulation data feeds the decision system. This behaviour is achieved through two synchronized agents—the real vehicle and its DT. The interface connecting these two agents with the simulation is depicted in Figure 7.

**Real Agent:** This agent processes input from a GNSS to create a localization pose within the real scenario. The actions from the Twin Agent, together with the localization data, are then fed into the operative level, which generates control commands, including the target linear velocity and target steering angle, which are sent to the Drive-by-Wire (DBW) module [43] at a frequency of 20 Hz. This module is responsible for translating target commands into electric signals to move the real vehicle at a frequency of 100 Hz. This is done using a PID controller for each target signal.

**Twin Agent:** The Twin Agent receives the vehicle’s location data, provided by the real-world localization module, and places the simulated vehicle at the same position but in the simulated environment. Meanwhile, the DM module processes the observations corresponding to the adversarial simulated vehicles and generates the corresponding actions, which are sent back to the Real Agent.

The synchronization between the real vehicle and CARLA is performed by updating the pose of the simulated ego vehicle with the latest localization data received from the real platform. The simulated adversarial vehicles remain fully controlled within CARLA, and their ground-truth positions and velocities are combined with the synchronized ego-vehicle pose to construct the observation vector used by the tactical DM module. Therefore, adversarial vehicles influence the real vehicle only through the high-level decisions generated from these simulated observations.

During PE, the surrounding adversarial vehicles are simulated in CARLA. Consequently, their positions, distances, and velocities are obtained from the simulator ground truth and synchronized with the real ego-vehicle pose. The real ego-vehicle state is provided by the localization module, while perception of real surrounding vehicles is outside the scope of this work.

## 6. Experiments

This section presents some quantitative results for each step of our CL methodology, highlighting the performance metrics achieved by our proposal. Additionally, visual qualitative results are available in our GitHub repository (https://rodrigogutierrezm.github.io/SENSORS2026curriculum.html, accessed on 7 June 2026).

Finally, an ablation study of our CL methodology is carried out, indicating the contribution of each stage to the overall system performance.

### 6.1. Results in SUMO

We conducted a study in SMARTS scenarios using the SUMO simulator to compare the various DRL methods evaluated in this work for the kinematic modelling of a vehicle. In this study, we focus on evaluating the agent’s learning of the first policy ($\pi_{j,s}$), which involves taking high-level actions, while the control signals are generated by the simulator. The operative level of our hybrid proposal is disabled. The agent’s performance is evaluated using the success rate, which is a straightforward indicator of the agent’s efficacy. Additionally, the average duration of an episode is calculated. The evaluation metrics are defined as follows:

- Success Rate (%): $success\;\left[\%\right]=\frac{n_{s}}{n_{e}}\times 100$
- Average Episode Time (s): $t_{avg}\;\left[s\right]=\frac{\sum t_{e}}{n_{e}}$

Here, $n_{e}$ represents the number of evaluation episodes, $n_{s}$ represents the number of success episodes, and $t_{avg}$ represents the average simulation time of an episode. To account for the uncertainty inherent in the probabilistic nature of the DRL policy, all continuous metrics (time, speed, jerk, and acceleration) are reported as mean ± standard deviation (μ±σ) over the testing episodes.

The comparison among DRL methods is included as a policy-selection step for the subsequent stages of the curriculum pipeline. It should not be interpreted as an algorithmic contribution of this work, since all evaluated methods are established algorithms.

#### 6.1.1. Comparison of DRL Methods

**Figure 8:**
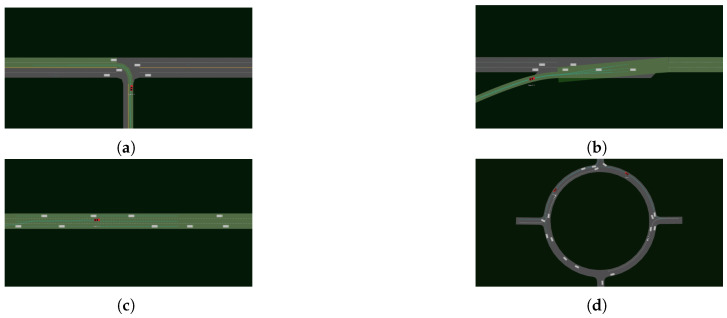
The designed scenarios within the SMARTS framework. (**a**) Unprotected left turn. (**b**) Three-lane merge. (**c**) Three-lane road. (**d**) Roundabout.

The ’Driving SMARTS 2022’ benchmark, launched in the NeurIPS 2022 Driving SMARTS competition [44], offers a variety of scenarios to evaluate DRL proposals for AD. We selectively focused on four scenarios, specifically targeting our urban-environment needs. A representation of these scenarios is presented in Figure 8.

In these scenarios, the traffic flow is consistently managed by the SMARTS simulator, with vehicles being generated at systematic intervals between one and three seconds and achieving maximum velocities of up to 15 m/s (54 km/h). This setup ensures a uniform testing environment across different driving situations, providing a controlled yet challenging context for evaluating the performance of our DRL proposal.

**Figure 9:**
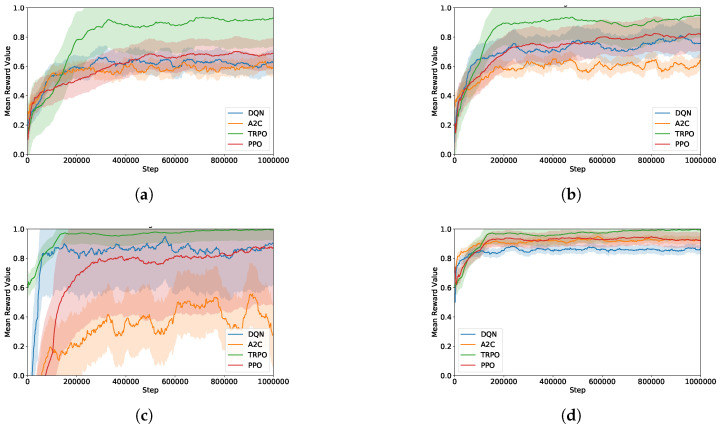
Evolution of the mean rewards during the training process for the DRL agents within the SMARTS framework: DQN (blue), A2C (orange), TRPO (green), and PPO (red). (**a**) Unprotected left turn. (**b**) Three-lane merge. (**c**) Three-lane road. (**d**) Roundabout.

Figure 9a illustrates the progression of the training process for the agents in the unprotected left-turn scenario. While the convergence point for all agents is comparable, a substantial disparity is observed in the average mean reward, with the TRPO agent achieving a higher mean reward. The DQN agent emerged as the fastest. Notably, the TRPO agent distinguished itself by achieving the highest success rate of 95.3% with a competitive average time, as shown in Table 1.

The progression of the training process in the three-lane merge scenario is depicted in Figure 9b. The TRPO agent emerges as the top performer, with the PPO and DQN agents demonstrating performances that are also competitive. The results reported in Table 1 show that the fastest agent is the A2C agent, with an average time of 5.61 s. However, the TRPO agent achieved the highest performance, showing a 98.4% success rate.

As shown in Figure 9c, in the three-lane road scenario, the maximum mean reward per episode is set slightly over 1 by the TRPO agent, which suggests its success in identifying an optimal policy. As depicted in Table 1, the TRPO agent achieves a remarkable success rate of 93.6%. Despite this, all four agents showed comparable average episode durations, with A2C being the quickest and the TRPO being the slowest.

A comparative analysis of training rewards in the roundabout scenario is presented in Figure 9d. The TRPO agent shows superior performance, characterized by a higher mean reward. All agents achieve convergence, typically around 200k time steps. During the testing phase, the results of which are presented in Table 1, the TRPO agent outperformed others, achieving the highest success rate of 91.7%. Conversely, the DQN exhibited the lowest success rate. Based on these results, TRPO is used in the following stages because it provides the most consistent performance across the selected scenarios.

#### 6.1.2. Global SOTA Comparison

We conduct training and testing for our TRPO agent, which was identified as the top performer among the proposed DRL algorithms. We compare the performance of our proposal in the selected SMARTS scenario with that of two global representative SOTA methods in the same scenario with its own POMDP formulation: ref. [21] employs a Transformer-based scene representation alongside an actor–critic DRL approach. Moreover, ref. [45] is based in three ingredients: expert demonstration, policy derivation, and DRL. The analysis is presented in Table 1.

In the three-lane merge scenario, our framework outshines, with a success rate of **98.40%**, surpassing the 96.00% achieved by [21] and excelling in a domain where ref. [45] provides no comparative data. Moreover, our framework’s performance is further evidenced in the roundabout scenario, recording a success rate of **91.70%**. This performance substantially exceeds the 76.00% success rate reported in [21] and the 84.00% reported in [45]. Efficiency, measured through the average completion time, further distinguishes our framework. Notably, in the three-lane merge scenario, it accomplishes the task in **21.9 s**, which is markedly quicker than the 28.60 s required by [21]. Although ref. [45] achieves a marginally higher success rate in the unprotected left-turn scenario, our framework maintains competitive success rates across all scenarios while consistently offering more efficient manoeuvre execution.

### 6.2. Results in CARLA

In this section, we evaluate the performance of our whole AD stack, which includes our hybrid DM architecture based on TRPO DRL, within the CARLA simulator to obtain a first version of the vehicle dynamics. Vehicle model training starts from the SUMO priors.

#### 6.2.1. Urban Scenarios for Reinforcement Learning

The urban scenarios simulate realistic, uncontrolled traffic conditions similar to the SUMO scenarios, where vehicles spawn every 3 to 5 s and move at speeds of 5 to 15 m/s (18 to 54 km/h). For lane-change scenarios, we use the *Town04* map in CARLA, which includes a 400 m road with four lanes. The intersection scenarios are set in the *Town03* map. Here, a roundabout scenario uses a 30 m radius roundabout; a merge scenario positions vehicles on a 60 m lane perpendicular to the ego vehicle’s path; and a crossroad scenario has two 50 m roads intersecting, with adversarial vehicles generated on both sides of the intersection. A detailed analysis is conducted on safety, comfort, and efficiency parameters that take into account the vehicle dynamics using the following metrics: the success rate (%), which measures the percentage of successful episodes and reflects safety; the 95th percentile of jerk (m/s^3^), indicating the smoothness of driving and passenger comfort; the maximum jerk (m/s^3^), which tracks the highest jerk experienced; the 95th percentile of acceleration (m/s^2^), representing the vehicle’s acceleration and its impact on comfort and efficiency; and the episode completion time (seconds), which measures the time taken to complete an episode and serves as an indicator of efficiency. While these metrics are sufficient to evaluate the primary goal of this work, validating the dynamic feasibility and comfort of sim-to-real transfer, we acknowledge important aspects they do not fully capture. Specifically, metrics such as interaction risk and strict rule compliance remain outside the scope of this study.

After the training process of the vehicle model over the aforementioned scenarios, we evaluate our proposal against the CARLA Autopilot [4]. This operates under the management of the Traffic Manager (TM) module, which has access to privileged, omniscient information about the environment and the state of all vehicles. In contrast to our agent, which relies on partial observations, the Autopilot uses this privileged data to generate trajectories avoiding collisions using a PID controller. Table 2 presents a comprehensive evaluation of our AD stack compared to the CARLA Autopilot across various testing driving scenarios: lane-change, roundabout, merge, and crossroad scenarios.

**Figure 10:**
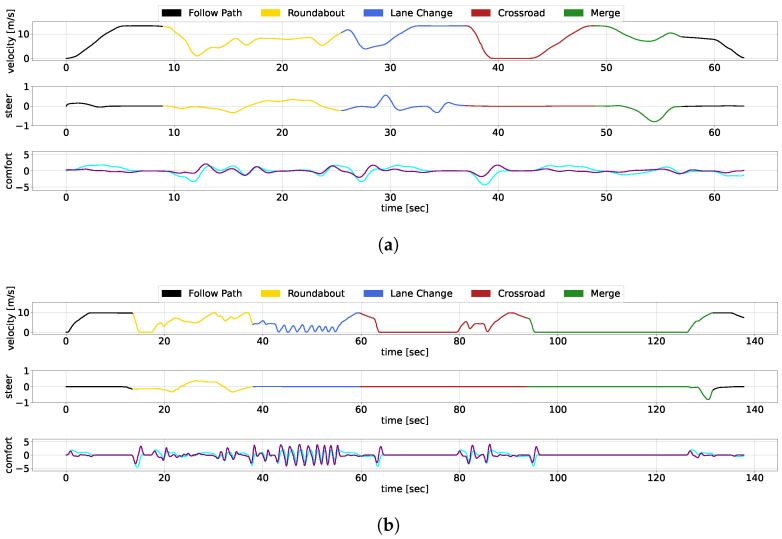
Temporal response of our AD stack (**a**) and the Autopilot (**b**) within a concatenated scenario. The linear velocity is depicted in the top chart, steering data is presented in the middle chart, and comfort metrics are illustrated in the bottom chart.

While the Autopilot achieves a success rate of 100% in all scenarios due to the centralized management carried out by the TM for all vehicles, as well as the access of the ego vehicle to the complete environmental data, our AD stack shows competitive performance, with high success rates in all scenarios, demonstrating its robustness and effectiveness. The jerk dynamics significantly favour our AD stack, with substantially lower 95th-percentile and maximum jerk values across all scenarios. This suggests a smoother and more comfortable ride under the tested conditions compared to the CARLA Autopilot, which exhibits higher jerk values. Acceleration dynamics further underscore our system’s efficiency, with our AD stack maintaining lower 95th-percentile acceleration in most scenarios, signifying gentler and potentially safer acceleration patterns. Our system completes scenarios in significantly less time than the CARLA Autopilot. Additionally, our AD stack maintains higher average speeds across scenarios. To provide a qualitative assessment of our proposal’s performance, we present the temporal response across the four concatenated scenarios, as illustrated in Figure 10.

#### 6.2.2. Digital Twins

In this step, we determine the physical parameters of our real vehicle and take measures of the real testing scenario to mimic a realistic configuration in CARLA to obtain our DT. In this work, the DT is defined as a calibrated digital replica of the ego-vehicle dynamics and the target merge scenario within CARLA. Its purpose is to reduce the dynamics-related discrepancy between simulation and real execution before the PE stage.

**Figure 11:**
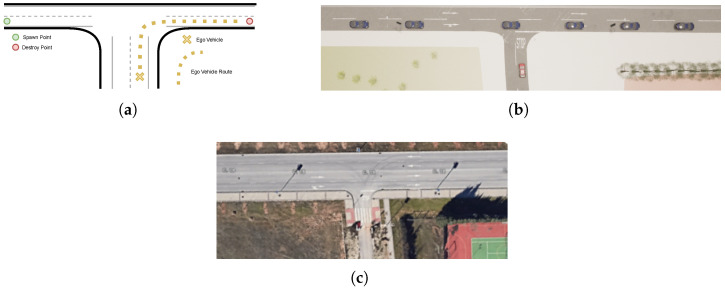
Merge intersection within the DT approach. (**a**) Visual representation of the traffic flow. (**b**) Bird’s-eye-view of the scenario in CARLA. (**c**) Bird’s-eye-view of the real-world intersection within our university campus.

To create the DT of our scenario, we use the facilities of our university campus. The process begins with acquiring the campus map from OpenStreetMap, which is imported into the RoadRunner tool [46] and subsequently into Unreal, allowing for the generation of the virtual environment within CARLA. While this map accurately represents the roads, it does not extend to environmental elements. Adversarial vehicles are generated on a lane perpendicular to the ego vehicle’s lane. These vehicles are subsequently destroyed when they reach the end of the scenario. A depiction of the scenario is presented in Figure 11.

To build our ego vehicle’s DT, we mimic the parameters of our real vehicle. The model is defined by a mass of 1030 kg, a maximum torque of 126 N·m, a drag coefficient of 0.60, a damping rate of 0.2, and a delay response of 0.50 s. Additionally, the vehicle has a maximum RPM of 5000, a moment of inertia (Moi) of 0.05 kg·m^2^, a tire friction of 0.85, and a maximum steer angle of 40 degrees. These calibrated parameters are intended to reduce the dynamics-related component of the RG rather than to model the complete sim-to-real discrepancy of autonomous driving. In particular, perception uncertainty and human-driver interaction variability are not explicitly modelled in this DT.

In this comparison, the “General” model corresponds to a policy trained in the generic CARLA merge scenario without DT fine-tuning. Therefore, it serves as a direct baseline to evaluate the effect of adapting the policy to the calibrated vehicle and environmental model.

Our AD stack is fine-tuned for this DT, yielding the following comparative metrics against the general vehicle model in CARLA described in the previous section. The results in Table 3 indicate that the AD stack fine-tuned for the DT consistently outperforms the general vehicle model. Notably, it achieves a higher success rate while reducing both jerk and acceleration, reflecting smoother and more controlled manoeuvres. Additionally, the DT agent completes the scenario slightly faster and at higher speeds.

### 6.3. Parallel Execution

For the PE testing experiments, our focus is on identifying the discrepancies between real and simulated signals. To this end, we execute identical scenarios using the DT only in CARLA and the PE with the Real and Twin agents. We explore three distinct traffic situations in the merge scenario, each differing in vehicle density and behaviour. For these experiments, the vehicle and environmental models defined in the DT stage are used directly. Although adversarial vehicles are simulated in the PE setup, they are not purely passive agents. As defined in Section 5.4, they follow different behavioural intentions, including yielding and non-yielding behaviours. Therefore, the ego vehicle is evaluated against controlled interaction patterns with different outcomes.

A quantitative comparison between the simulated DT and real vehicle performance under varying traffic conditions during a PE is presented in Table 4. Overall, the results show a strong correspondence between simulation and real-world behaviour, confirming the reliability of the DT in replicating the dynamics. The DT consistently exhibits slightly superior performance in terms of motion smoothness and efficiency. In low traffic flow, both the simulated and real vehicles achieved a 100% success rate, with minimal differences in time (19.18 s vs. 19.99 s) and jerk (1.34 m/s^3^ vs. 1.78 m/s^3^). Under mixed and high traffic flows, success rates remained above 95% in all cases, while the maximum jerk and acceleration values of the real vehicle were only marginally higher—typically within 10–20% of the simulated values. Although execution times increased with traffic density, this trend was consistent across both environments.

**Figure 12:**
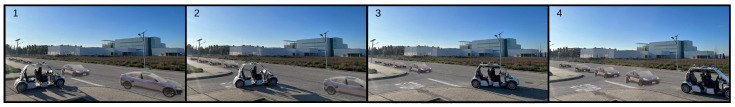
Parallel execution of the mixed-traffic-flow merge scenario. (**1**) Ego starts moving. (**2**) Ego stops to yield. (**3**) Ego merges into the intersection. (**4**) Ego reaches the end of the scenario.

**Figure 13:**
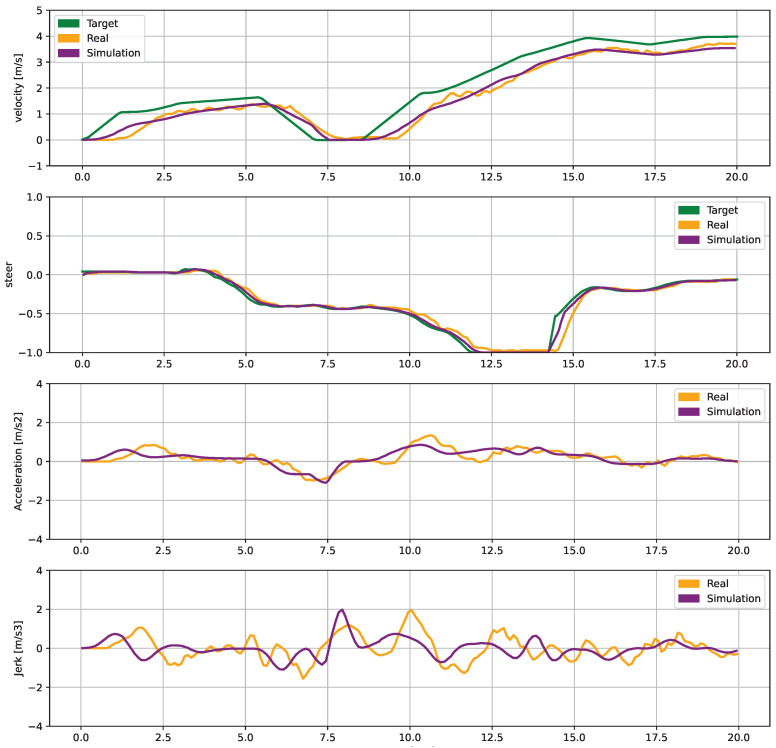
Control signals during a parallel execution: Identical signals are provided to the real and simulated vehicles. The target, real, and simulated signals are illustrated.

**Figure 14:**
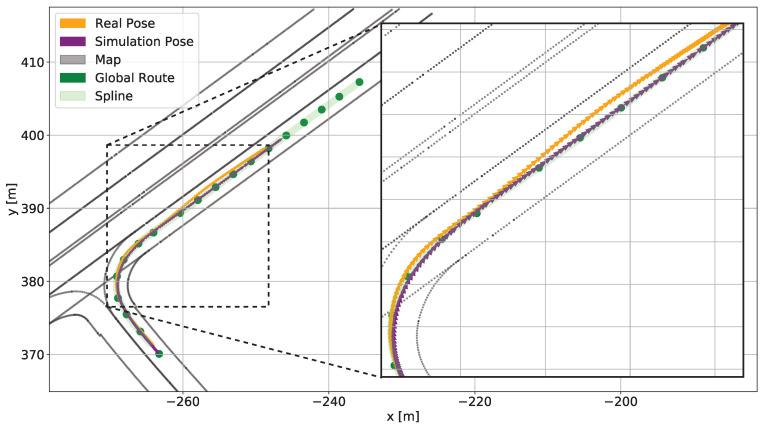
Trajectory-level validation during parallel execution in the merge scenario. The simulated digital twin follows the nominal trajectory, while the real vehicle shows a slight deviation.

For a qualitative analysis, we present an example of the mixed-flow use case. In this scenario, the ego vehicle initiates its movement and, due to the presence of adversarial vehicles, prompts the DRL agent to select the stop action, leading to a reduction in velocity until the vehicle stops. Once a gap is identified, the drive action is selected, causing the velocity signal to increase until it reaches the nominal velocity set by the operative level. The vehicle then merges behind an adversarial vehicle and follows it. In this scenario, the vehicle response shows a visible delay after changes in the target signal, yet the jerk and acceleration signals remain comparable between the real and simulated responses. This behaviour is mainly associated with the physical execution layer, since the target velocity generated by the operative level must be tracked by the drive-by-wire system, which introduces actuator dynamics and PID tracking delays. Minor discrepancies may also arise from localization uncertainty, synchronization effects, and surface irregularities. A visual representation of this experiment is depicted in Figure 12, while the control signals are shown in Figure 13. Additionally, Figure 14 presents a trajectory-level comparison between the simulated DT and the real vehicle during the same PE experiment. The simulated vehicle follows the nominal reference trajectory more closely, as expected in the absence of real actuation delays, localization noise, and surface irregularities. The real vehicle shows a small deviation from the ideal path but remains consistent with the simulated trajectory throughout the manoeuvre.

### 6.4. Ablation Study and Contribution of Each Curriculum Stage

This ablation study is included to quantify the contribution of each stage of the curriculum pipeline and to assess how the staged transfer process affects sim-to-real performance. Since this work focuses on a real-world merge scenario, the study will be carried out exclusively for this case. The analysis begins with an ablation study of the training phases. To evaluate our curricular approach, we must take into account that three different training sessions were executed: training in SUMO (in the three-lane merge Scenario), training in CARLA using the prior SUMO model (in the *Town03* merge scenario), and training using our DTs from the prior model in CARLA (in our simulated campus scenario). Additionally, we estimate the results of training our DT from scratch without the curriculum approach. The outcomes of the experiments, validated in the CARLA DT with 100 episodes, are depicted in Table 5.

As we can see, the prior model trained in SUMO reduces its performance when tested in the CARLA DT, primarily because this simulator takes into account the dynamics of the vehicles. Training in the *Town03* scenario and testing in the CARLA DT results in competitive performance, with an 88.30% success rate. However, the best results are obtained through the fine-tuning process of the vehicle and environmental models, achieving success rates similar to those observed in previous sections—specifically, a 91.80% success rate and a similar or better average time. Conversely, the adoption of a curriculum learning strategy involving pre-training in SUMO and subsequent fine-tuning in CARLA accelerates model convergence by a factor of 67 relative to training from scratch. For these comparisons, the stopping criterion was defined as the stabilization of the success rate over a 50-episode window on the specified hardware.

While the advantages of our curriculum methodology regarding training efficiency are evident, a comprehensive evaluation of how each curriculum phase impacts the final real-world validation is essential. To assess this impact, we conduct an ablation study examining the PE testing in the campus merge scenario. We evaluate the impact of each stage by analysing discrepancies between real and simulated control signals under randomized traffic conditions in our real-world merge scenario with PE. Each configuration corresponds to different combinations of our curriculum steps: (1) training only in SUMO; (2) training only in CARLA, which represents a general CARLA-trained policy evaluated without DT fine-tuning; (3) sequential training in SUMO and CARLA; (4) fine-tuning in the DT using a model pre-trained in CARLA without the SUMO priors; and (5) the complete curriculum approach, i.e., SUMO → CARLA → DT using the priors of the previous steps.

We quantify the fidelity and performance of each approach using the following metrics:

- Mean Normalized Cross-Correlation (MNCC) [47]: To assess the similarity between control signals from simulation and the real world, we compute the MNCC for velocity, steering, acceleration, and jerk.
- Decision Consistency (%): This metric measures the alignment of high-level decisions between simulation and reality throughout the episode. It is defined as follows:
  (20) $$\mathrm{Consistency}=\frac{N_{\mathrm{match}}}{N_{\mathrm{total}}}\times 100,$$
  where $N_{\mathrm{match}}$ is the number of high-level actions that match between simulated and real executions and $N_{\mathrm{total}}$ is the total number of decisions.
- Success Rate (%): This metric captures the percentage of successful episodes over the total number of test episodes, as presented in Section 6.1.
- Training Time (h): We define the total training time as the sum of hours required to reach convergence across all phases in a configuration.

Table 6 summarizes the performance of each configuration across 100 testing episodes under different traffic conditions.

SUMO-only training achieves the lowest training time (5 h) but underperforms in both low-level signal similarity and high-level behaviour, achieving a decision consistency of only 67.5% and a success rate of 20%. Conversely, CARLA-only training yields slightly better signal alignment and consistency (69.5%) but incurs a very high computational cost (1650 h). Combining SUMO and CARLA significantly improves the success rate to 40% while keeping the training time low (21.5 h), highlighting the benefits of using SUMO for efficient initial learning (kinematic model) and CARLA for realism (dynamic model). Adding the DT phase further boosts performance. The configuration with CARLA + DT reaches a high decision consistency of 94.6% and a 95% success rate but with the same high training time (1650 h). The full curriculum (SUMO + CARLA + DT) achieves the best overall performance, with the highest signal fidelity (MNCC >0.97), the highest success rate (100%), and near-perfect decision consistency (94.8%), all with a relatively low training cost of just 24.75 h.

In summary, incorporating SUMO in the early training phases significantly reduces convergence time. CARLA, on the other hand, enables smoother transitions between curriculum stages and allows for testing in real scenarios. Ultimately, the complete curriculum—combining SUMO, CARLA, and DT—achieves the best alignment with real-world behaviour while keeping a low overall training cost.

### 6.5. Comparison with State-of-the-Art Frameworks

While the ablation study analyses the internal contributions of our methodology, it is crucial to situate our approach within the broader landscape of AD. However, a direct quantitative comparison is infeasible, as third-party algorithms cannot be simply deployed on our specific hardware setup. Therefore, Table 7 presents a qualitative comparison focusing on the training strategy and the scope of real-world execution.

First, regarding Eend-to-end Imitation Learning (IL), approaches like CIL [48] and TransFuser [49] achieve high performance across many scenarios but require massive expert datasets and heavy computational resources. In contrast, our curriculum-based DRL is significantly more lightweight, learning efficient policies through interaction, without the need for terabytes of labelled driving data. Furthermore, while IL shows broad scenario coverage, real-world validation is often limited; for instance, CIL was validated on a 1/5-scale truck with low interaction, while representative methods like TransFuser rely primarily on simulation leaderboards.

Second, regarding direct sim-to-real RL, works such as Wayve [50] present impressive results by employing DL directly on a real vehicle. However, this approach relies on exhaustive domain randomization or risky real-world training and is currently limited to simpler tasks like lane following without complex agent interaction. Other RL approaches, such as CIRL [51], remain confined to simulation. In contrast, our strategy employs a fine-tuned DT, allowing the agent to adapt to our vehicle’s dynamics (friction and actuation delays) faster and more precisely than brute-force randomization, enabling complex logic beyond lane keeping.

Finally, regarding rule-based systems like RSS [52] or POMDP solvers [53], we found that while they offer formal safety guarantees, they predominantly present results in simulation, with a lack of reported deployment on full-scale vehicles under the noisy conditions of our target scenarios.

A key distinction of our work lies in the validation safety and execution quality. By utilizing simulated adversaries, we validate critical collision avoidance behaviours without physical risk. Consequently, our system is one of the few to demonstrate smooth and safe manoeuvring at an intersection in a closed-loop real-world environment.

## 7. Conclusions and Future Works

We developed a hybrid Decision Making architecture for real-world-oriented scenarios, following a Curriculum Learning methodology to reduce the dynamics-related component of the reality gap. This approach involves an initial training phase in a lightweight simulator (SUMO) to model vehicle kinematics; the use of a Digital Twins in a highly realistic simulator (CARLA) for fine-tuning of the model; and, finally, testing of the complete AD stack on a real vehicle with Augmented Reality (AR) observations through Parallel Execution. This methodology allows us to simulate complex scenarios while reducing the safety and economic limitations inherent to real-world experimentation. In the present study, the proposed approach is evaluated across several urban driving scenarios in simulation, while the real-world validation is restricted to a controlled merge scenario. Therefore, the conclusions regarding real-vehicle execution should be interpreted within the scope of this tested scenario rather than as a complete real-world validation across all urban driving scenarios.

Although the results support the effectiveness of the proposed staged transfer pipeline, the present work primarily addresses the dynamics-related component of the reality gap, including vehicle dynamics, actuation response, and scenario geometry. Perception uncertainty, sensor noise, and complex interaction with real human drivers are not explicitly modelled in this study and remain important directions for future work.

In future work, we plan to extend the parallel execution approach to the rest of the urban scenarios studied in simulation in this work, with special attention to roundabouts and multi-directional intersections. Additionally, we will test with real vehicles acting as adversaries, incorporating vehicle-to-vehicle systems instead of relying solely on simulation ground-truth data. We also aim to scale our proposal by including more diverse scenarios with different layouts, traffic patterns, and vehicle types, as well as to experiment with real sensors, transitioning from simulated inputs to actual hardware to assess the performance and reliability of the perception modules under real-world conditions. Finally, exploring alternative optimization paradigms, such as genetic algorithms and particle swarm intelligence, constitutes a valuable line of research. Future work will also extend the evaluation protocol with direct interaction-safety metrics, including minimum time to collision, post-encroachment time, time headway, minimum distance, hard-braking events, rule violations, lane invasion, and the near-collision rate.

## Figures and Tables

**Table 1:** Results in SUMO (SMARTS). Comparison between DRL methods and global SOTA proposals in the SMARTS scenarios across 1000 testing episodes for each scenario. The success rate (sr) in percentage and average time (at) in seconds are presented.

Scenario	Comparison of DRL Methods								Global SOTA Comparison			
	DQN		A2C		TRPO		PPO		[21]		[45]	
	sr ↑	at ↓	sr ↑	at ↓	sr ↑	at ↓	sr ↑	at ↓	sr ↑	at ↓	sr ↑	at ↓
Unprotected left turn	88.5	**11.13**	94.6	11.76	**95.3**	12.22	93.3	12.81	94.0	12.50	**96.0**	14.26
Three-lane merge	82.1	5.81	82.8	**5.61**	**98.4**	21.9	86.7	7.18	96.0	28.60	-	-
Three-lane road	83.4	17.12	81.2	**16.32**	**93.6**	24.34	88.3	17.92	-	-	-	-
Roundabout	81.5	16.12	89.9	13.97	**91.7**	37.47	90.1	**12.45**	76.0	56.60	84.0	**36.62**

**Table 2:** Results in CARLA. Comparison between our agent and Autopilot across 1000 testing episodes for urban scenarios. Metrics include success rate (sr), 95th and max jerk, 95th acceleration, time, and speed. Metrics are reported as mean ± SD.

Metric	Lane Change		Roundabout		Merge		Crossroad	
	Ours	Autopilot	Ours	Autopilot	Ours	Autopilot	Ours	Autopilot
sr [%] ↑	91.20	**100**	95.10	**100**	96.40	**100**	87.90	**100**
95th Jerk (m/s^3^) ↓	**1.58** ± 0.32	9.12 ± 2.15	**1.73** ± 0.35	5.93 ± 1.84	**2.67** ± 0.51	3.63 ± 0.95	**1.83** ± 0.42	14.6 ± 3.20
Max Jerk (m/s^3^) ↓	**5.64** ± 1.12	13.56 ± 3.50	**2.20** ± 0.55	12.16 ± 2.90	**3.83** ± 0.82	9.98 ± 2.45	**2.16** ± 0.48	22.8 ± 4.10
95th Accel. (m/s^2^) ↓	**1.53** ± 0.15	3.65 ± 0.55	**1.61** ± 0.18	2.67 ± 0.42	2.53 ± 0.22	**2.51** ± 0.38	**1.55** ± 0.14	3.88 ± 0.61
Time (s) ↓	**68.94** ± 4.25	128.56 ± 8.40	**20.32** ± 1.85	30.23 ± 3.15	**25.83** ± 2.10	34.16 ± 4.20	**23.14** ± 1.95	38.84 ± 5.10
Speed (m/s) ↑	**9.05** ± 0.85	3.61 ± 0.45	**5.83** ± 0.62	5.45 ± 0.70	**2.45** ± 0.35	1.92 ± 0.28	**4.26** ± 0.48	0.89 ± 0.15

**Table 3:** Results in CARLA. Comparison between the general vehicle model agent trained in the merge scenario and the DT agent across 1000 testing episodes. Both are evaluated using the DT vehicle model within the merge DT scenario. Metrics include the success rate (SR), 95th-percentile and maximum jerk, 95th-percentile acceleration, time, and speed. Metrics are reported as mean ± SD.

Metric	Merge DT Scenario	
	General	Digital Twin
sr [%] ↑	88.30	**91.80**
95th Jerk (m/s^3^) ↓	3.58 ± 0.45	**3.09** ± 0.38
Max Jerk (m/s^3^) ↓	3.64 ± 0.52	**3.12** ± 0.41
95th Acceleration (m/s^2^) ↓	3.53 ± 0.35	**2.44** ± 0.22
Time (s) ↓	20.33 ± 2.10	**19.98** ± 1.85
Speed (in m/s) ↑	2.34 ± 0.30	**2.85** ± 0.25

**Table 4:** Parallel execution. Comparison between the simulated digital twin and the real vehicle during the parallel execution in the merge scenario under different traffic conditions, across 100 testing episodes per condition. Metrics include success rate (sr), 95th and max jerk, 95th acceleration, time, and speed. Metrics are reported as mean ± SD.

Metric	Low Traffic Flow		Mixed Traffic Flow		High Traffic Flow	
	Simulation (DT)	Real (PE)	Simulation (DT)	Real (PE)	Simulation (DT)	Real (PE)
sr [%] ↑	**100**	**100**	98.0	95.0	99.0	98.0
95th Jerk (m/s^3^) ↓	**1.34** ± 0.12	1.78 ± 0.21	1.73 ± 0.25	1.98 ± 0.32	1.36 ± 0.15	1.43 ± 0.22
Max Jerk (m/s^3^) ↓	**1.96** ± 0.20	2.01 ± 0.28	2.02 ± 0.31	2.43 ± 0.45	2.05 ± 0.28	2.08 ± 0.35
95th Acceleration (m/s^2^) ↓	**0.98** ± 0.08	1.54 ± 0.15	1.52 ± 0.18	1.86 ± 0.24	1.11 ± 0.12	1.32 ± 0.19
Time (s) ↓	**19.18** ± 1.10	19.99 ± 1.55	35.76 ± 4.20	39.23 ± 5.10	53.76 ± 6.50	55.82 ± 7.20
Speed (m/s) ↑	**4.97** ± 0.35	4.06 ± 0.42	2.36 ± 0.55	2.11 ± 0.60	1.19 ± 0.15	1.08 ± 0.18

**Table 5:** Ablation study of the curriculum methodology. Comparison of training approaches in terms of success rate (sr), average time per episode (at), number of episodes to converge (ec), and total training time (tt). All experiments were conducted on an NVIDIA RTX 3090 (12 GB VRAM).

Metric	Training SUMO	Training CARLA	Fine-Tuning CARLA	From Scratch
sr [%]	75.60	88.30	91.80	94.60
at (s)	21.53	20.33	19.98	19.96
ec	1 M	1 M + 10 K	1 M + 15 K	1 M
tt (h)	5	21.5	24.75	1650

**Table 6:** Ablation study of the curriculum methodology. Comparison of different curriculum configurations for parallel-execution validation in the merge scenario. Metrics include signal similarity (MNCC for velocity, steering, acceleration, and jerk), high-level decision consistency, the success rate across 100 evaluation episodes, and the total training time.

Phase	Velocity MNCC ↑	Steering MNCC ↑	Acceleration MNCC ↑	Jerk MNCC ↑	Decision Consistency (%) ↑	Success Rate (%) ↑	Training Time (h) ↓
SUMO Only	0.765	0.782	0.643	0.514	67.5	20	5
CARLA Only	0.774	0.789	0.671	0.555	69.5	35	1650
SUMO + CARLA	0.747	0.790	0.685	0.579	70.3	40	**21.5**
CARLA + DT	0.977	0.981	0.927	0.873	94.6	95	1666.5
SUMO + CARLA + DT	**0.978**	**0.988**	**0.930**	**0.879**	**94.8**	**100**	24.75

**Table 7:** Comparison of our approach vs. state-of-the-art frameworks.

Paradigm	Reference	Scenario	Sim-to-Real Strategy	Training Source	Real-World Execution
End-to-End IL	CIL [48]	Urban Navigation	Data Augmentation	Offline Expert Data	Yes (1/5 Scale Truck)
	TransFuser [49]	Complex Urban	Visual Perturbations	Offline Expert Data	None (CARLA Leaderboard)
Direct RL	Wayve [50]	Lane Following	Domain Randomization	Real-World Driving	**Yes (Automated Vehicle)**
	CIRL [51]	Urban Navigation	Feature Control	High-Fidelity Simulators	None (Simulation)
Rule-Based	RSS [52]	Safety Critical	Parameter Tuning	Manual Design	None (Formal Model)
	Hubmann [53]	Intersections	Model Calibration	Hand-crafted Rules	None (Simulation)
Ours	Proposed	Intersections	Digital Twin	Curriculum	**Yes (Automated Vehicle)**

## Data Availability

The original contributions presented in this study are included in the article. Further inquiries can be directed to the corresponding author.
